# Assessing the drivers and solutions of green innovation influencing the adoption of renewable energy technologies

**DOI:** 10.1016/j.heliyon.2024.e30158

**Published:** 2024-04-24

**Authors:** Yasir Ahmed Solangi, Rakan Alyamani, Cosimo Magazzino

**Affiliations:** a Renewable Energy Lab, College of Engineering, Prince Sultan University, Riyadh, 11586, Saudi Arabia; b Department of Political Science, Roma Tre University, Rome, Italy

**Keywords:** Green innovation, Economic growth, Renewable energy technologies, Sustainable development, AHP, SAW

## Abstract

The degradation of the environment in China is accelerating along with economic expansion. Adoption of renewable energy technologies (RETs) is crucial for reducing the adverse impacts of economic growth on the environment and fostering sustainable development. This study attempts to identify the green innovation drivers and sub-drivers that affect the adoption of RETs in China and provide solutions for boosting their implementation. The study prioritized the drivers, sub-drivers, and strategies of green innovation by combining the Analytical Hierarchy Process (AHP) and Simple Additive Weighting (SAW) methods. In the study, the triple bottom line (TBL) approach has been used to determine the economic, societal, and environmental driving forces. The study also suggests strategies for encouraging the use of RETs. The results of the AHP method revealed that economics is the most crucial driver, with a weight of 0.376, followed by environmental (0.332), and social (0.291) drivers. The findings of the SAW method indicated that government green innovation initiatives, consumer initiatives, and industry initiatives are the most significant strategies for deploying RETs in China. This study has important theoretical and practical ramifications for encouraging China to adopt RETs. The suggested approaches can help researchers, business professionals, and policymakers promote sustainable development in China.

## 1 Introduction

Sustainable development has emerged as a global development paradigm [1]. Since the start of the twenty-first century, green technology innovation has been a crucial component of the global industrial revolution and technical competitiveness [2,3]. Around the world, numerous countries have created policies to promote innovation in green technology. One of the top economies in the world, China places a strong emphasis on green innovation to address the problems brought on by climate change and global warming. Climate change is one of the biggest worldwide issues, affecting ecosystems, economy, and society [4]. Climate change, air pollution, and resource depletion are threatening sustainable development worldwide. Moreover, one of the main global contributors to greenhouse gases (GHGs) is CO_2_ emissions, which has accelerated global warming [5]. In order to achieve sustainable economic and social growth, natural resources and environmental circumstances have a significant impact on any country's development [6]. China has progressed toward ecologically sustainable growth through legislation, standards, and investments to decrease pollution, conserve resources, and foster green innovation. It basically involves developing and spreading renewable energy technology (RETs), products, and services that reduce environmental consequences and boost economic growth and social welfare [7,8]. Green innovation is seen as a significant driver of sustainable development in China since it may alleviate environmental issues and create new economic opportunities [9]. Moreover, green innovation relies on RETs to decrease pollution, save resources, and promote sustainable development.

There is an extensive empirical literature on the green innovation, its determinants, and impacts on climate change mitigation and adaptation efforts and sustainable development in China. For instance Ref. [2], explore how does government policy affects Green Technology Innovation (GTI) in China. The study concludes heterogeneous direct and indirect impacts government policies on GTI. Another study [10] determine that technological level and industrial structure directly affect carbon emission in Chinese provinces. In comprehensive planning cities, a spatial self-correlation study of resource-based cities shows that government assistance and influencing variables like industrial structure and economic growth favor green innovation. Environmental rules, on the other hand, have a negative influence and stifle urban green innovation. As a result, it is critical to strike a balance between these elements when developing policies to support green innovation in resource-constrained communities [11]. find inertial development and self-reinforcing mechanism among digitalization, technological innovation and green economic development (GED). The study explored positive promotion impact of digitalization on GED. Whereas, the impact of technological innovation on latter is insignificant. Moreover, the study also found positive impact of GED on technological innovation in short run. However, this impact gradually declines to zero in the long run [12]. evaluate the link between green innovation, resource efficiency and sustainable growth in E7 economies including China. The study finds positive association between green innovation and resource efficiency. The findings of [13] show a substantial geographical clustering pattern of green technology innovation level and carbon intensity across Chinese regions. GTI and carbon intensity in a local area have an Inverted-U shape, with low levels of green technology innovation tending to promote carbon emissions. However, as levels of green technology innovation increase to a certain threshold, this relationship shifts to an inhibiting effect on carbon emissions. The previous study examined renewable energy sustainability in 27 EU nations using a composite assessment approach [14]. The model used the DPSIR, RAGA, projection pursuit, and fuzzy clustering iteration. The model considers energy, economy, society, and environment. The findings indicated that Denmark and Sweden have the most sustainable energy in the EU. Besides, another related study, the authors evaluated the potential of China's shale gas industry for sustainable development [15]. The outcome indicated that core technological capability is the biggest sustainable development challenge. Similar study, conducted the empirical analysis of Sichuan and Chongqing to analyze the sustainability of shale gas industry [16]. In the earlier work, the scholars focused on the quantitative assessment of renewable energy in several European countries during 2007–2016 [17]. The Germany, France, and Italy outperformed than other European countries.

Green innovation is essential to attaining sustainable development and tackling environmental issues. However, RETs must be used to realize the benefits of green innovation. The adoption of such technologies is influenced by various factors that impact the decision-making processes of many stakeholders, including governments, industry, civil society, and consumers. As indicated by its many patent filings and institutional policies aiming to raise green technology innovation levels, China is one of the top countries in green technology innovation [2,18]. RET adoption remains challenging due to policy complexity, delay, executive force, and policy initiative success. Solving this problem requires investigating RET adoption's green innovation drivers. Addressing drivers' priorities first assists in enhancing green technologies and environmental sustainability. Further research is required to determine how regulations affect RET adoption in China and the drivers of green innovation. It can assist determine which policies work best to promote green technology in China's industries and regions.

The primary focus of current study is to examine the drivers of green innovation influencing the adoption of RETs in China based on the theoretical Triple Bottom Line (environmental, social, and economic factors). A study by Ref. [19] attempted to examine the drivers of green innovation based on the TBL foundations using NARDL on quarterly data from Pakistan economy. Since it is important to examine the association between the TBL and green innovation [20], the current study examine the drivers of green innovation to adopt RETs in the perspective of theoretical ground provided by TBL. This study prioritizes the economic, social, and environmental green innovation drivers of RET adoption in China. The study also highlights green innovation strategies that influence RET adoption. Therefore, to achieve these objectives, the study integrates multi-criteria decision analysis (MCDA) methodologies i.e. Analytical Hierarchical Process (AHP) and Simple Additive Weighting (SAW) to asses prioritize the drivers of green innovation and strategies to implement RETs in China.

## 2 Drivers and strategies of green innovation for RETs in China

### 2.1 Drivers and sub-drivers of green innovation

The adoption of green innovation and RETs are becoming increasingly important in order to address environmental issues such as pollution, resource depletion, and climate change. The criteria or elements that affect adopting RETs are referred to as drivers. In the study, the various key drivers and sub-drivers of green innovation are identified for further assessment. The drivers and sub-drivers of green innovation for RETs in China are summarized in Table 1.

**Table 1:** Drivers and Sub-drivers of green innovation.

Driver	Sub-driver	Brief description	Reference
**Economic drivers (A)**	Cost-effectiveness (A-1)	It refers to the ability of an RETs to provide a cost-effective solution compared to outdated energy technologies. In this regard, the expected financial returns of the investment in the technology, the costs associated with the use and maintenance of the technology and the costs associated with the inputs or materials required to produce the technology are important determinants of green innovation.	[[21], [22], [23], [24], [25]]
	Financial incentives (A-2)	Refers to government programs or incentives that fund RET development and adoption. Technology development and use are supported by government subsidies and tax incentives. Besides, green financing also boosts green innovation.	[22,23,26,27]
	Competitive advantage (A-3)	It refers to the ability of a company to gain a competitive edge in the market by adopting RETs. Furthermore, the green innovation enables the companies to differentiate their products and introduce green products.	[19,22,23]
	Access to funding (A-4)	The governments are allocating the financial resources support the development and adoption of RETs. The policies are also devised to enable the ability to access investment from venture capitalists or other investors to support the development and adoption of the technology.	[[27], [28], [29], [30]]
**Social drivers (B)**	Public awareness (B-1)	It refers to the level of knowledge and understanding of environmental issues among the general public. In this regard, the knowledge of environmental issues and their impact on their health impacts the public behavior to demand for eco-friendlier products and clean environment.	[24,25,31]
	Social responsibility (B-2)	The level of social responsibility that companies and individuals feel towards the environment and society is also one of the social drivers to stimulate green innovation. In this regard, corporate social responsibility shapes the culture of innovation. Furthermore, companies and individuals take into account the ethical considerations while making decisions about the adoption of RETs.	[25,32,33]
	Consumer preferences (B-3)	The consumers' preferences regarding the product features and design, convenience and ease of use of eco-friendly products also encourage the green innovation in the economy. Furthermore, the product quality and performance also affect the consumer behavior to demand for eco-friendly product.	[25,28,34]
	Stakeholder engagement (B-4)	It refers to the level of engagement and collaboration with stakeholders such as NGOs, civil society groups, and local communities. The extent to which companies collaborate with NGOs and civil society groups to address environmental issues, communication and engagement with local communities, and participation in industry associations that promote RETs may also drive green innovation.	[35,36]
**Environmental drivers (C)**	Air pollution (C-1)	The negative impacts of air pollution on the environment and public health. The ability of RETs to reduce emissions of air pollutants, enabling the use of alternative energy sources to reduce dependence on fossil fuels and reduce air pollution are the major drivers of green innovation.	[4,28,37]
	Resource depletion (C-2)	It refers to the depletion of natural resources and the need to promote resource efficiency. Since the green innovation provides the opportunities to use resources of clean energy, purification of water, and natural resource conservation, such benefits of the former provide strong reasons to believe that it is should be promoted to ensure sustainability.	[12,38,39]
	Climate change (C-3)	It refers to the negative impacts of climate change on the environment and society. Firms are under pressure to decrease their carbon footprints due to various factors such as the rise in global warming, climate change, and demands from stakeholders including investors, employees, and network partners.	[19,39]
	Waste management (C-4)	It refers to the management of waste and the need to promote circular economy. Green technology and innovation has been making possible the recycling and reusing waste materials.	[40,41]

The analysis of the TBL approach i.e., economic, social, and environmental drivers of green innovation and their sub-drivers would assist policymakers and industry practitioners identify viable solutions to the constraints that impede the adoption of RETs and encourage long-term growth in China.

### 2.2 Strategies to stimulate adoption of RETs

**Fig. 1:**
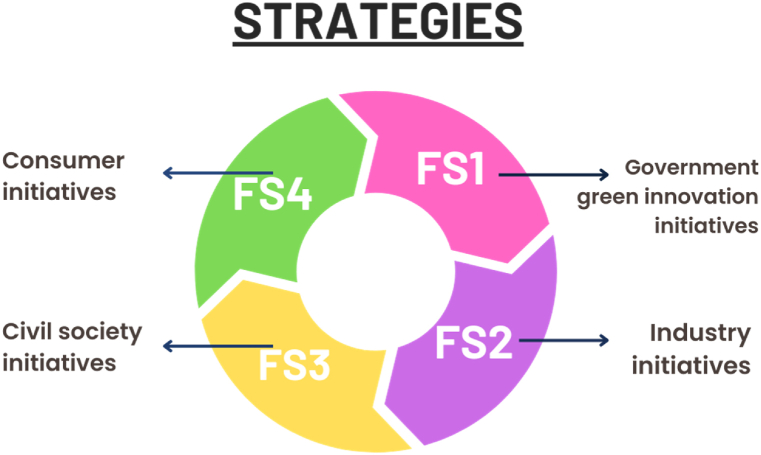
The proposed strategies for the implementation of RETs.

In this study, the several key strategies are proposed for the sustainable adoption of RETs and foster green innovation in China. The government of China has already implemented specific policy plans to promote the zero-carbon in the country, although the further regulations are required to effectively adopt green practices. Fig. 1 displays the identified vital strategies of this study.

The following four have been identified as significant strategies to implement RETs in China.

#### 2.2.1 Government green innovation initiatives to promote RETs adoption (FS1)

To promote RET adoption, the Chinese government should establish green innovation policies. The government can highlight policies and tactics to persuade business decision-makers to consider green technology innovation [2,42]. The government should implement RETs regulations and standards. For its adoption, the country can provide subsidies, financial incentives, and tax exemptions. Moreover, the provision of enabling infrastructure for RET adoption can help achieve green technology economic goals.

#### 2.2.2 Industry initiatives to stimulate adoption of RETs (FS2)

Industrial businesses need to recognize the value of green innovation and how RETs may help maintain development and provide them a competitive edge [43,44]. Industries can work with stakeholders and other firms to promote RETs since cost-effectiveness and efficiency can be improved by R&D.

#### 2.2.3 Civil society initiatives to stimulate adoption of RETs (FS3)

The air pollution and climate change are major environmental challenges in China. These issues are addressed by government programs that support green growth and a low-carbon transition. Therefore, the NGOs, social companies, and grassroots actions may also help implement RETs [45]. These programs can raise awareness, mobilize social resources, provide technical assistance, and increase demand for eco-friendly products and services.

#### 2.2.4 Consumer initiatives to stimulate adoption of RETs (FS4)

Strategy is essential for China's RET adoption and sustainable growth. Sustainable lifestyles and green activities encourage acceptance of these technologies, while green product and service demand drives innovation and investment. So, the government, society, and consumers must effort collectively to foster the adoption of these technologies in the country.

## 3 Methodology

**Fig. 2:**
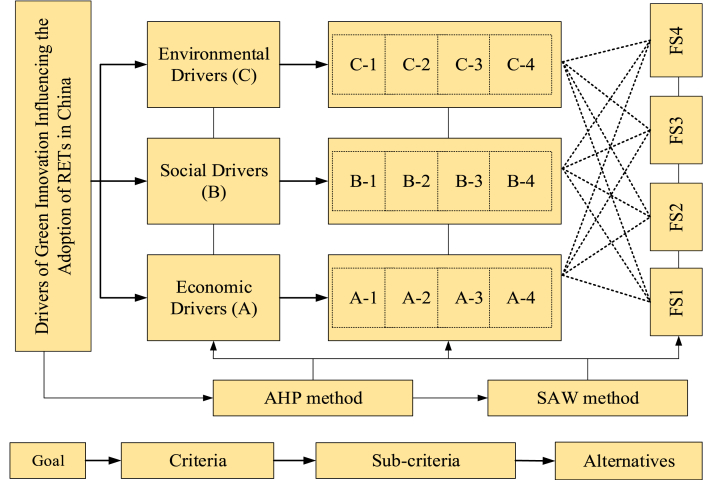
The hierarchical-based methodology.

The MCDA based decision-making framework has been adopted to analyze the problem of this study. Thus, the AHP and SAW methods are utilized to assess and rank the drivers, sub-drivers, and strategies of green innovation for implementing RETs in China. The AHP method is used to evaluate the drivers and sub-drivers of green innovation. Later, the SAW method is used to rank the most significant strategies for adopting RETs. Fig. 2 shows the decision-making framework of this study.

### 3.1 AHP method

The AHP method was developed by Saaty in 1970s [46]. The AHP is a transparent approach, which allows decision to incorporate their preferences and judgments into the decision-making process [47]. This approach is a simple and effective tool for handling complicated situations containing both objective and subjective assessments. This method employed when several decision-makers must select from a wide range of contradictory options while dealing with ambiguity and certainty. AHP is widely used method in various fields such as healthcare [48], mining [49], supply chain [50], agriculture [51], and education [52]. This approach is hierarchical-based that assess the various criteria and sub-criteria using pairwise comparison matrix and arrive at well-suited decision [53]. Therefore, the pairwise comparison matrix are used to determine the relative importance of each criterion over other criterion. The AHP method involves the following steps [54].

Step 1 Identify criteria and sub-criteria

Step 2 Develop a hierarchical-based decision-making structure.

Step 3 Develop pair-wise comparisons matrix. Table 2 shows the Saaty's 1–9 points scale.

**Table 2:** Saaty's 1–9 points scale.

Numerical value	Description
1	Equal importance
3	Moderate importance
5	Strong importance
7	Very strong importance
9	Absolute importance
2,4,6,8	Intermediate values

Step 4 Compute the Consistency Index (CI):

In this step, CI can be used to measure the consistency of the pair-wise comparison of the matrix. CI can be presented as [55].

(1) $$CI=\frac{\lambda_{\max}-n}{n-1}$$

here $\lambda_{\max}$ is the eigenvalue and n is the no. of main criteria

Step 5 Computing the Consistency Ratio (CR)

(2) $$CR=\frac{CI}{RI}$$

Where RI is the random consistency index, which is presented in Table 3. During the pair-wise comparison, the CR must be within the limit of 0.1; if it exceeds above 0.1, then the results could be inconsistent [56].

**Table 3:** Random consistency index.

n	RI
1	0.00
2	0.00
3	0.058
4	0.90
5	1.12
6	1.24
7	1.32
8	1.41
9	1.45
10	1.49

### 3.2 SAW method

The SAW method enables decision-makers to examine and compare alternatives using multiple criteria. The SAW method assigns a weight to each criterion based on its importance and scores each alternative based on its performance [57]. This purpose of this method is to find the sum of the weighted performance rating for each alternative on all attributes [58]. This method has been used in various disciplines such as energy [59], supply chain [60], and education [61]. Table 4 provides the scale of attributes used in the study.

**Table 4:** Attribute scale.

Number	Attribute
1	Very Low
3	Low
5	Medium
7	High
9	Very High

The SAW method involves the following steps [62]:

Step 1. Construct the decision matrix, including *n* decision criteria:

(3) $$A=\left(\begin{array}{cccc} a_{11} & a_{12} & \cdots & a_{1n}\\ a_{21} & a_{22} & \cdots & a_{2n}\\ \vdots & \vdots & \cdots & \vdots \\ a_{m1} & a_{m2} & \cdots & a_{mn} \end{array}\right)={\left[a_{ij}\right]}_{m\times n}$$

where a_ij_ is the value of alternative i with respect to decision criterion j.

Step 2. Normalize the decision matrix by the following equations:

(4) $$\mu_{ij}=\frac{1}{1+{\left(\frac{a_{ij}}{f_{1}}\right)}^{-f_{2}}},i=1,2,\ldots ,m,j=1,2,\ldots n$$

(5) $$\mu_{ij}=\frac{1}{1+{\left(\frac{a_{ij}}{f_{1}}\right)}^{f_{2}}},i=1,2,\ldots ,m,j=1,2,\ldots ,n$$

Step 3. Determine weights of decision criteria by subjective or objective weighting methods. The resulting weight vector would be as follows:

(6) $$W=\left[w_{1},w_{2},\ldots ,w_{n}\right]$$

where n is the number of decision criteria or evidence maps.

Step 4. Compute significance degree of each alternative by the following equation:

(7) $$S_{i}=\sum_{i=1}^{m}w_{j}\mu_{ij},i=1,2,\ldots ,m$$

In the study five experts were consulted to analyze the decision making. The consulted experts were well-skilled and professional consists of environmental analyst, academia, economic analyst, and industry practitioner. All these experts were contacted via webmail and they were asked to provide their useful insights in determining the drivers, sub-drivers, and strategies for promote green innovation and for adoption of RETs.

## 4 Results and discussion

In this research, the AHP and SAW methods has been used to assess and prioritize the drivers and strategies of green innovation for implementing the RETs in China. Therefore, in the subsequent sub-sections, the detailed findings are provided and discussed.

### 4.1 Ranking of drivers using AHP method

Table 5 shows the ranking of main-drivers of green innovation. The findings indicated that, economic (A) is the most influential in driving the adoption of RETs for green innovation in China, followed by environmental (C) and social drivers (B), respectively. This indicates that economic benefits such as cost savings, revenue generation, and market competitiveness are the primary motivation for firms to adopt green technologies, while environmental and social benefits are also considered important factors. Therefore, encouraging the widespread implementation of RETs in diverse economic sectors require laws and programs that offer financial incentives and support for green innovation.

**Table 5:** The ranking of drivers for promoting the adoption of RETs.

Drivers	Weight	Rank
Economic (A)	0.376	1
Social (B)	0.291	3
Environmental (C)	0.332	2

### 4.2 Results of sub-drivers using AHP method

#### 4.2.1 Prioritization of economic sub-drivers

**Fig. 3:**
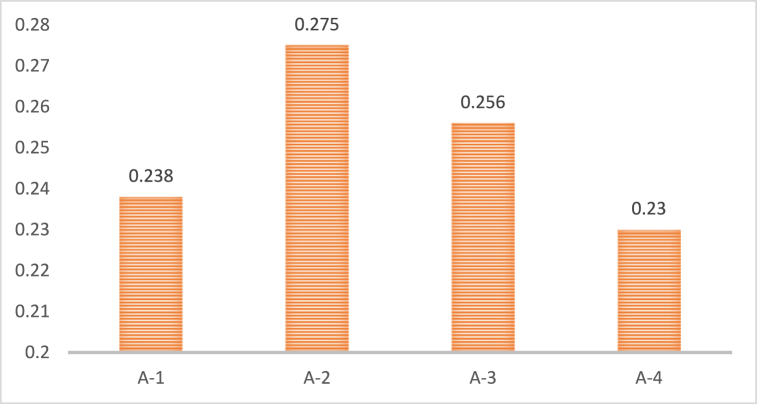
The ranking of economic sub-drivers.

Fig. 3 shows the final ranking of economic sub-drivers. The findings show that the financial incentives (A-2) are the most significant sub-driver. It is because due to the fact that these factors offer the innovators direct and immediate advantages and lower the risks and uncertainties of green innovation. Financial incentives come in many shapes and sizes, including subsidies, tax credits, grants, loans, and awards. The second most significant sub-driver is a competitive advantage (A-3), which represents the potential and long-term advantages that green innovation may provide innovators regarding market share, reputation, consumer loyalty, distinctiveness, or quality [19,22,23]. Offering superior or distinctive goods and services that satisfy consumers' requirements and expectations regarding the environment, society, and culture can give businesses a competitive edge. The third most significant sub-driver is cost-effectiveness (A-1), measures green innovation's efficacy and financial viability. Reduced manufacturing, operation, maintenance, or disposal costs of the goods or services and higher revenues from sales, licensing, or royalties can increase cost-effectiveness. Regulations, standards, or pricing are outside influences that can impact cost-effectiveness. The least significant sub-driver is access to funds (A-4) [28,29], which refers to the availability and accessibility of financial resources for green innovation. Thus, the access to finance may be impacted by the type and stage of innovation, the scale of the inventor, the sector, the sources and qualifying standards for funding, or the institutional and legal frameworks.

#### 4.2.2 Prioritization of social sub-drivers

**Fig. 4:**
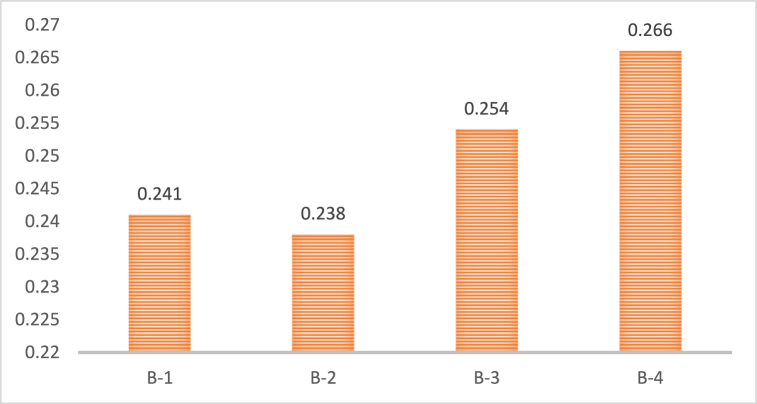
The ranking of social sub-drivers.

Fig. 4 displays the final ranking of social sub-drivers. The rankings of the sub-drivers of social drivers of green innovation show that stakeholder participation (B-4) is the most crucial sub-driver, followed by consumer preferences (B-3), public awareness (B-1), and social responsibility (B-2). Understanding stakeholders' concerns and objectives through stakeholder engagement allows organizations to develop environmentally sustainable products and services [35,36]. Because it increases customer demand for environmentally friendly products and services, public education is essential. Companies may use a variety of tactics to inform and educate the public about the benefits of sustainability, including social media campaigns and advertising. Consumer preferences, which control the demand for eco-friendly products and services, are essential for fostering green innovation. Businesses may develop eco-friendly goods that satisfy the requirements and tastes of their customers by researching consumer preferences [25,28,34]. Social responsibility, which demands firms accept responsibility for their influence on the environment and society, is another essential sub-driver of green innovation [25,32]. It may require reducing waste, environmental effect, and sustainable methods. Public awareness affects customer demand for eco-friendly products and services [24,25]. Therefore, companies may educate consumers about sustainability through social media and advertising.

#### 4.2.3 Prioritization of environmental sub-drivers

**Fig. 5:**
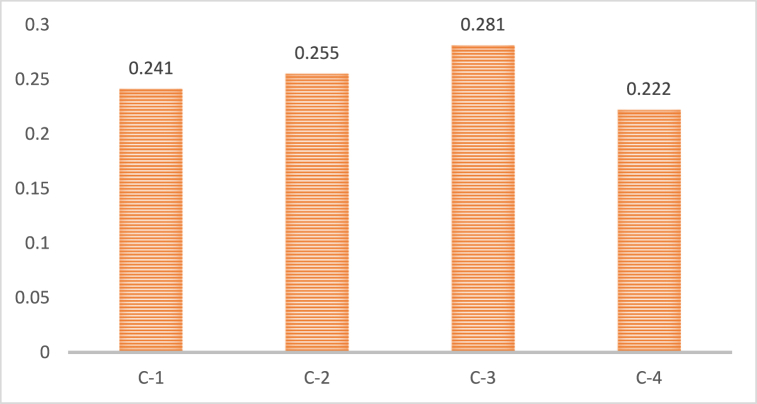
The ranking of environmental sub-drivers.

Fig. 5 depicts the final ranking of environmental sub-drivers. Based on their significance for attaining sustainable development, the sub-drivers of environmental drivers of green innovation were ranked. The findings indicate that the most crucial sub-driver is climate change (C-3), which is then followed by resource depletion (C-2), air pollution (C-1), and waste management (C-4). The TBL theoretical framework [63], empirical data from various industries and nations, and the possible benefits and downsides of green innovation for each sub-driver may be used to analyze these rankings. The TBL is a decision-making approach that considers environmental, social, and economic issues. According to the rankings, tackling climate change is critical for attaining environmental sustainability, whereas resource depletion need to be addressed for economic sustainability. Furthermore, as they considerably influence human health and well-being, air pollution and waste management are crucial for attaining social sustainability [40]. Empirical facts from many industries and regions also support these rankings. For example, the transportation industry contributes heavily to climate change [10], and the development of electric cars has the potential to cut GHG emissions.

Moreover, addressing air pollution may require stricter legislation and improved enforcement [37], whereas trash management may require more extensive public education and behavior change. Finally, the rankings of environmental drivers of green innovation sub-drivers give a valuable framework for evaluating the relative relevance of each sub-driver in attaining sustainable development. According to the findings, climate change is the most crucial sub-driver of green innovation since it substantially challenges global sustainability. By creating renewable energy sources, increasing energy efficiency, and adopting carbon capture and storage technology, green innovation can assist in alleviating the harmful effects of climate change [11,43,[64], [65], [66], [67], [68]]. Another key driver of green innovation is resource depletion, which emphasizes the need to use natural resources more effectively and sustainably. Green innovation enhances resource efficiency [12] by inventing goods that consume fewer resources, using renewable resources, and implementing circular economy models. Air pollution is a critical environmental and public health concern that may be addressed through green innovation by creating clean energy and transportation technologies, imposing emission limits, and encouraging sustainable urban planning [69]. Despite receiving the lowest ranking, waste management is nevertheless essential for achieving environmental sustainability since it lowers waste production, promotes recycling and composting, and develops cutting-edge waste treatment technologies.

### 4.3 Prioritization of strategies using SAW method

The SAW approach effectively prioritizes various strategies based on their relative value for a particular goal. The goal in this situation is to encourage the use of RETs in China. Table 6 present the final ranking of four feasible strategies for adoption of RETs in China.

**Table 6:** The final ranking value of strategies promoting the adoption of RETs.

Strategy	Value	Rank
Government green innovation initiatives (FS1)	0.974	1
Industry initiatives (FS2)	0.613	3
Civil society initiatives (FS3)	0.521	4
Consumer initiatives (FS4)	0.918	2

According to the findings, the government green innovation initiatives (FS1) are ranked first, followed by consumer initiatives (FS4), industry initiatives (FS2), and civil society initiatives (FS3). This ranking indicates that the government may have a major impact on the market and plays a crucial role in promoting the usage of RETs in China through its policies and activities. China has instituted institutional mechanisms to increase the levels of green technology innovation and has recently placed a greater emphasis on the role that innovation in green technologies plays in achieving sustainability. The influence of policies on green technology innovation, on the other hand, varies depending on their number, efficacy, and execution [18].

The government can use RET subsidies, tax exemptions, and limits, among other policy measures, to encourage people and corporations to use RETs. For instance, the government can offer tax benefits to people who install RETs in their houses, or it can subsidize businesses that make investments in energy-efficient technologies. These laws may improve the environment for the implementation of RETs and reduce the entrance barriers for environmentally conscious businesses. Governments may provide financial support in the form of subsidies to encourage RETs. Rather of penalizing polluters for their emissions, subsidies offer compensation for reducing emissions. Subsidies include things like grants, low-interest loans, tax breaks, and procurement laws. Subsidiaries have been used to achieve a wide range of goals, such as cleaning up contaminated land, preventing erosion through agricultural grants, lending low-interest money to small farmers, encouraging land conservation through grants, and funding the recycling of commercial, industrial, and residential products. Subsidies, like taxes, encourage the reduction of emissions but require market action in order to be eligible [25,70]. Furthermore, the market for green technology products may grow as a result of government measures promoting corporate responsibility and raising public awareness of ecologically responsible consumption.

Therefore, if there is focused governmental support and a positive industrial development environment, company innovation ability may be increased in supporting green technology innovation. However, other studies contend that environmental rules may impede the effectiveness of technological innovation and lower firm productivity, as demonstrated by empirical assessments [2,71]. It is notable that the subsidy programs by the government may have a "crowding effect," which hinders businesses from developing new green technologies. However, specific assistance measures have increased the business's ability to develop new goods. However, businesses may prioritize the development of new technologies and products that produce economic advantages without more targeted legislation to boost green technology innovation. It might reduce the vitality of enterprise innovation in fostering the development of green technologies [72]. Consumer efforts are placed second in significance, indicating the importance of developing public awareness and fostering sustainable purchasing practices. Businesses can employ a variety of strategies, such eco-labeling and green marketing, to tell and educate consumers about the benefits of RETs and the ways in which their choices will affect the environment. Customers can also actively support companies that promote sustainability by endorsing greener goods and services. The importance rankings for industry and civil society activities are third and fourth, respectively. Business sector initiatives to develop and advance RETs, including as research and development, technology transfer, and green supply chain management, are examples of industry activity.

## 5 Conclusion

Using the TBL theoretical framework, which integrates environmental, social, and economic issues, the current study intends to analyze the drivers of green innovation impacting the adoption of RETs in China. Given the significance of understanding the link between TBL and green innovation, this research aims to identify and prioritize the economic, social, and environmental drivers of green innovation that impact the adoption of RETs in China. Furthermore, the study identifies and prioritizes policies and initiatives that encourage green innovation in implementing RETs. In this regard, this study adopted a MCDA that combines AHP and SAW techniques to achieve these goals.

The research indicates that economic drivers are the most significant drivers of sustainability, followed by environmental and social factors. The most important economic sub-drivers for green innovation are financial incentives and competitive advantage. Moreover, cost-effectiveness and access to funding are other critical economic sub-drivers for green innovation. Policymakers can use this information to develop effective strategies for promoting green innovation and accelerating the transition to a sustainable economy. Among the social sub-drivers of green innovation to adopt RETs, stakeholder engagement has been ranked as the most crucial factor. Consumer preferences, public awareness, and social responsibility have been ranked 2nd, 3rd, and 4th, respectively. Regarding environmental sub-drivers, the research has identified climate change as the most critical sub-driver for green innovation, followed by resource depletion, air pollution, and waste management. These findings can help policymakers prioritize their efforts to address these environmental challenges and promote the adoption of RETs.

Various limitations to this study must be considered. The study focuses solely on adopting RETs in China, with little consideration given to the worldwide consequences of encouraging green innovation. Future studies should look at the possibility of cross-country collaborations to enhance global sustainable development. Furthermore, the study does not mention the ethical and societal ramifications of encouraging RETs. More studies might investigate the ethical and societal aspects of fostering green innovation. In addition, the research may differ based on the technology or sector under consideration. Future studies might investigate the relative significance of the drivers and sub-drivers for various technologies and sectors. Moreover, the limitations and assumptions of the AHP and SAW methodologies utilized in this study must be acknowledged. Future studies might look into other decision-making techniques to find effective green innovation strategies. Despite these limitations, this study provides a valuable framework for understanding the drivers of green innovation and has the potential to contribute to long-term growth.

## Availability of data and material

The data will be available on request.

## CRediT authorship contribution statement

**Yasir Ahmed Solangi:** Writing – review & editing, Writing – original draft, Validation, Methodology, Formal analysis, Data curation, Conceptualization. **Rakan Alyamani:** Writing – review & editing, Visualization, Validation, Resources. **Cosimo Magazzino:** Writing – review & editing, Visualization, Investigation, Funding acquisition.

## Declaration of competing interest

The authors declare that they have no known competing financial interests or personal relationships that could have appeared to influence the work reported in this paper.
